# *Lacticaseibacillus rhamnosus* LM1019 Enhances Natural Killer Cell Activity in Defined Adult Subsets: A Double-Blind, Placebo-Controlled Trial

**DOI:** 10.3390/life15071002

**Published:** 2025-06-24

**Authors:** Jeseong Park, Yeji You, Sung-Hwan Kim, Hye-Jin Ku, Hye-Jung Han, Chul-Hong Kim, Tae-Rahk Kim, Minn Sohn, Inah Kim, Chaemin Baeg

**Affiliations:** 1 Microbiome R&D Center, Lactomason Co., Ltd., Jinju 52840, Republic of Korea; yjyou@lactomason.com (Y.Y.); trkim@lactomason.com (T.-R.K.); ms@lactomason.com (M.S.); 2 Food Research Center, Binggrae Co., Ltd., Namyangju 12253, Republic of Korea; ksh1224@bing.co.kr (S.-H.K.); hj-ku@bing.co.kr (H.-J.K.); hyejung725@bing.co.kr (H.-J.H.); cms51@bing.co.kr (C.-H.K.); 3 Global Medical Research Center, Seoul 06526, Republic of Korea; inah@gmrc.co.kr (I.K.); cm3690@gmrc.co.kr (C.B.)

**Keywords:** probiotics, *Lacticaseibacillus rhamnosus*, immune enhancement, dietary supplements, immunosenescence, clinical trial

## Abstract

Probiotics offer a non-pharmacological approach to support immune function, yet clinical evidence for strain-specific benefits remains limited. We conducted an 8-week, randomized, double-blind, placebo-controlled trial of *Lacticaseibacillus rhamnosus* LM1019 in 121 generally healthy adults. Both the active and placebo arms produced comparable within-group increases in natural killer (NK) cell cytotoxicity and modest, non-differential declines in circulating cytokines; safety and tolerability were excellent, with mild adverse events evenly distributed. In a post-hoc subgroup defined by age ≥ 40 years, baseline white blood cell count ≥ 5.0 × 10^3^/µL, and LDL cholesterol < 130 mg/dL, the probiotic arm demonstrated statistically significant enhancements in NK activity (*p* = 0.021–0.008 across all effector-to-target ratios), whereas no change was observed in the placebo group. These findings suggest that this intervention may selectively boost NK-mediated immunity in individuals with preserved baseline immune and lipid profiles. Future larger trials using phenotype-driven enrollment and controlled dietary intake are warranted to confirm and extend these results.

## 1. Introduction

Aging entails a progressive decline in immune competence—immunosenescence [1]—characterized by thymic involution with reduced naïve T-cell output and contracted T-cell receptor diversity [2,3,4], accelerated telomere shortening after mid-life [5,6], and accumulation of senescent CD8^+^CD28^−^CD57^+^ T-cells [7,8]. These shifts become pronounced after age 40, as telomere erosion and homeostatic proliferation further skew lymphocytes toward memory phenotypes [9,10]. In parallel, B-cell naïve output and affinity maturation wane [11,12], while innate immunity deteriorates—NK-cell cytotoxicity and neutrophil chemotaxis decline, and a low-grade inflammatory milieu (“inflammaging”) marked by elevated interleukin-6 (IL-6), tumor necrosis factor-α (TNF-α), and C-reactive protein (CRP) emerges [13,14,15]. Collectively, these changes impair pathogen clearance, weaken vaccine responses, and elevate risks of infection, autoimmunity, and malignancy in older adults [16,17,18,19,20], a vulnerability underscored by the COVID-19 pandemic and the urgent need for safe, non-pharmacologic interventions to bolster host defense [21,22,23].

Probiotics—live microorganisms that confer health benefits when administered in adequate amounts—have garnered interest as one such non-drug approach due to their reshaping of gut microbial ecology, enhancing of short-chain fatty acid production, and engagement of gut-associated lymphoid tissues to modulate cytokine profiles and lymphocyte trafficking [24,25,26,27,28,29]. NK cells, which mediate early antiviral and antitumor defense via target-cell lysis and IFN-γ/TNF-α secretion [30,31,32], have shown enhanced activity following probiotic supplementation in several clinical trials: *Weissella cibaria* JW15 increased NK cytotoxicity and improved cytokine profiles over eight weeks [33]; yogurt fermented with *Lactiplantibacillus plantarum* HOKKAIDO enhanced NK activity in younger adults [34]; and *Bifidobacterium animalis* ssp. *lactis* HN019 improved NK-cell and polymorphonuclear function in the elderly [35,36]. Meta-analytic evidence further supports these strain-specific immunostimulatory effects across diverse human cohorts.

Among probiotic strains, *Lacticaseibacillus rhamnosus* GG stands out for its robust mucosal adhesion and well-characterized immunomodulatory properties [37], consistently enhancing or preserving NK cytotoxicity in older adults when combined with soluble corn fiber or *B. animalis* BB-12 [38,39]. Our candidate strain *Lacticaseibacillus rhamnosus* LM1019 (LM1019)—isolated from Korean infant feces—exhibits strong in vitro proteolytic activity, favorable metabolic effects in vivo [40,41], and potent immune stimulation, including macrophage cytokine induction and restoration of NK, CD4^+^ T-cell, and IFN-γ levels in immunosuppressed mice [42].

We therefore conducted an 8-week, randomized, double-blind, placebo-controlled trial to test whether daily LM1019 supplementation could (1) enhance NK-cell cytotoxicity as a primary endpoint and (2) favorably modulate circulating cytokines, white blood cell counts, and fatigue severity as secondary outcomes in adults with mildly reduced immune parameters, with pre-specified subgroup analyses to identify phenotypes most responsive to intervention.

## 2. Materials and Methods

### 2.1. Study Design

**Figure 1:**
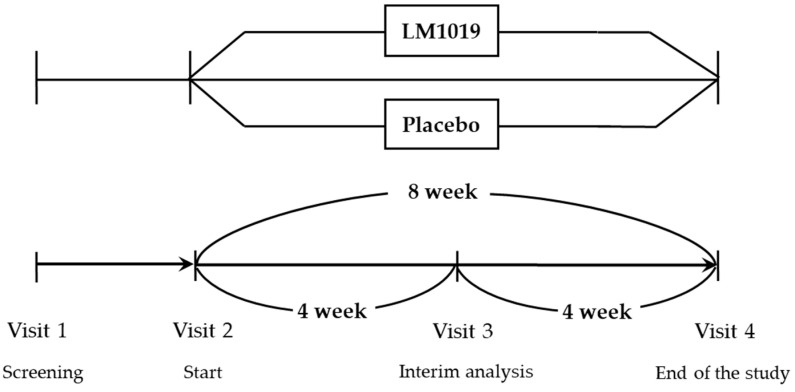
Schematic diagram illustrating the study design.

Figure 1 is a schematic diagram of this clinical trial, illustrating the study design. Participants who voluntarily signed the informed consent form underwent comprehensive screening (visit 1), which included demographic assessments, lifestyle surveys (including the Perceived Stress Scale (PSS)) [43], reviews of medical and medication history, physical examinations, vital sign measurements (blood pressure, pulse, and body temperature), anthropometric measurements (height, body mass index (BMI), weight), clinical pathology tests, pregnancy tests for women of childbearing potential, and evaluations for the occurrence of upper respiratory tract infections, stomatitis, herpes zoster, simple cystitis, and WBC count. Eligible participants meeting the inclusion and exclusion criteria were then randomly assigned to either the test group or the control group in a 1:1 allocation ratio.

Participants consumed the assigned product—either the test product or placebo—for the entire 8-week study period. Efficacy assessments were conducted at the 4-week mark and again at the end of the 8-week supplementation period. The final efficacy assessment at 8 weeks served as the primary endpoint for evaluating the overall efficacy and safety of the intervention in this clinical trial.

### 2.2. Clinical Study

#### 2.2.1. Inclusion and Exclusion Criteria

The study was conducted at the Global Medical Research Center in Seoul, Korea, under the supervision of an institutional review board (IRB) with the approval code GIRB_22726-JI. The clinical trial was also registered with the Clinical Research Information Service (KCT0009746). All participants provided written informed consent before starting the study.

Participants were randomly assigned 1:1 to receive either LM1019 (1.5 × 10^9^ CFU/day) or an identical placebo for 8 weeks. Both the LM1019 and placebo capsules were manufactured by Lactomason Co., Ltd. (Jinju, Republic of Korea). The placebo consisted of maltodextrin as the sole inactive ingredient and was encapsulated in the same gelatin capsule—identical in size, color, weight, and taste—as the LM1019 capsules, ensuring both participants and investigators remained blinded to group assignment. Participants were instructed to maintain their usual lifestyle and diet throughout the 8-week study—including habitual intake of kimchi and other traditional fermented dishes—and to avoid altering eating patterns. Any restrictions related to probiotic supplements or fermented dairy products were already covered under the exclusion criteria. Both participants and investigators remained blinded to the group assignments throughout the study. Participants were also advised to maintain consistent lifestyle habits, avoiding alcohol, strenuous exercise, and significant dietary changes.

#### Inclusion Criteria

Eligible participants must have met all of the following at screening (visit 1):

1. Age between 19 and 75 years.
2. White blood cell counts between 3.0 and 8.0 × 10^3^ cells/µL.
3. History of ≥2 upper respiratory tract infections (e.g., common cold, pharyngitis, rhinitis) within the past 12 months.
4. History of ≥2 episodes of stomatitis or herpes zoster within the past 12 months.
5. Recurrent uncomplicated cystitis: ≥3 episodes in the past year, or ≥2 episodes in the past 6 months.
6. Perceived Stress Scale (PSS) score ≥ 16.
7. Willingness and ability to maintain usual diet, avoid probiotic-rich supplements at ≥4×/week, and comply with all study procedures.
8. Signed informed consent.

#### Exclusion Criteria

Participants meeting any of the following at screening (visit 1) were excluded:

1. Chronic or uncontrolled medical conditions (autoimmune disease, active malignancy, uncontrolled hypertension [SBP ≥ 140 mmHg or DBP ≥ 90 mmHg], diabetes with fasting glucose ≥ 126 mg/dL, or thyroid dysfunction [TSH < 0.1 or < 10 µIU/mL]).
2. Recent vaccination (influenza, COVID-19, herpes zoster) within 3 months, or COVID-19 infection within 6 weeks prior to visit 1.
3. Use of immunosuppressants, systemic steroids, antibiotics, or acid suppressants within 1 month prior to visit 1.
4. Regular (≥4×/week) consumption of probiotic supplements or probiotic-rich fermented dairy products in the month prior to visit 1.
5. Severe gastrointestinal disorders (e.g., active ulcer, inflammatory bowel disease).
6. Pregnancy, breastfeeding, or planning pregnancy during the study (unless using effective contraception).
7. Participation in another interventional clinical trial within 8 weeks prior to visit 1 or planned participation during this study.
8. Known allergy to probiotics or capsule excipients (e.g., maltodextrin).
9. Body mass index ≥ 30 kg/m^2^.
10. Excessive alcohol intake (>210 g/week for men, >140 g/week for women) within 1 month prior to visit 1.
11. Any other condition that, in the investigator’s judgment, could compromise safety or protocol compliance.

### 2.3. Outcome Measures

#### 2.3.1. Efficacy Assessment

All NK cell cytotoxicity assays were performed strictly according to the manufacturers’ protocols. Peripheral blood mononuclear cells (PBMCs) were isolated from EDTA-anticoagulated whole blood by density-gradient centrifugation using Lymphoprep™ (StemCell Technologies, Vancouver, Canada; Cat. No. 07851) exactly as instructed by the supplier. NK cells were then purified with the MojoSort™ Human NK Cell Isolation Kit (BioLegend, San Diego, CA, USA; Cat. No. 480054) following the manufacturer’s step-by-step protocol, and purity was confirmed by flow cytometry.

Isolated NK effector cells and K562 target cells (ATCC, Manassas, VA, USA) were co-cultured in Corning 96-well round-bottom plates (Corning, Corning, NY, USA) at the specified E:T ratios (50:1, 25:1, 12.5:1) for 4 h at 37 °C in a 5% CO_2_ incubator (Heracell VIOS, Thermo Fisher Scientific, Waltham, MA, USA). Control wells were set up exactly as described in the Promega CytoTox 96^®^ protocol, including target-only (spontaneous LDH release), lysis control (maximum LDH release), and medium-only (background).

After incubation, plates were centrifuged and supernatants transferred to flat-bottom plates for LDH quantification using the CytoTox 96^®^ Non-Radioactive Cytotoxicity Assay (Promega, Madison, WI, USA; Cat. No. G1780), with every step—from reagent preparation to incubation times—carried out per the Promega user manual. Absorbance at 492 nm was read on a Sunrise™ microplate reader (Tecan, Männedorf, Switzerland). Cytotoxicity (%) was then calculated using Promega’s recommended formula:

$$Cytotoxicity(\%)=\frac{Experimental\;Release-Spontaneous\;Release}{Maximum\;Release-Spontaneouse\;Release}\times 100$$

In this formula, we find the following:

- Experimental Release refers to LDH levels measured in wells containing NK cells co-cultured with K562 target cells.
- Spontaneous Release refers to LDH levels measured in wells containing target cells alone.
- Maximum Release refers to LDH levels measured in wells containing target cells treated with lysis solution to achieve complete cell lysis.

Secondary outcomes included changes in serum cytokine levels (IL-1β, IL-2, IL-6, IL-12(p70), IFN-γ, TNF-α), WBC count, and Fatigue Severity Scale (FSS) scores. Serum samples were thawed, centrifuged (ZIPocrit-12, Shaking Inc., Seoul, Republic of Korea), and assayed using the MILLIPLEX^®^ MAP Human Cytokine/Chemokine Magnetic Bead Panel (Merck Millipore, Burlington, MA, USA; Cat. No. HCYTOMAG-60K) in accordance with the manufacturer’s protocols. All incubations and washes were performed on a handheld magnetic block (Life Technologies, Carlsbad, CA, USA). Beads were read on a MAGPIX^®^ system (Luminex Corporation, Austin, TX, USA) and analyzed with xPONENT^®^ v4.2 software; concentrations were interpolated from standard curves supplied by the manufacturer.

All NK cell cytotoxicity and cytokine assays were performed at U2Bio Co., Ltd. (Seoul, Republic of Korea) under standardized SOPs. Hematology, blood chemistry, and urinalysis were conducted at Seegene Medical Foundation (Seoul, Republic of Korea). Vital signs (height, weight, blood pressure, pulse, body temperature) were measured on-site at the Global Medical Research Center (Seoul, Republic of Korea).

Assessments occurred at baseline (visit 2, week 0) and post-intervention (visit 4, week 8). Secondary outcomes also included the frequency of upper respiratory tract infections (URTIs) and participants’ perceived health improvement, evaluated via structured surveys at weeks 4 and 8 (visits 3 and 4). All data were analyzed for within- and between-group differences using appropriate statistical models.

#### 2.3.2. Safety Assessment

The evaluation was conducted by reviewing the frequency and severity of adverse events recorded in individual participant adverse event logs, as well as by assessing abnormal findings from clinical pathology tests (hematological, blood chemistry, and urine tests), vital signs (blood pressure, pulse, and body temperature), and anthropometric measurements (weight). Abnormal values from clinical pathology tests and abnormal findings in vital signs that were deemed clinically significant were documented in the individual adverse event logs within the case report form and were further evaluated through statistical analysis.

### 2.4. Statistical Analysis

All statistical analyses were performed using SAS^®^ (Version 9.4, SAS Institute, Cary, NC, USA). For handling missing data, the last observation carried forward method was used, replacing missing values with the most recent data available, except for baseline values, which were not used as replacements. Missing data for all other analysis items, except efficacy variables, were analyzed without imputation.

Descriptive statistics, including mean and standard deviation, were calculated for the data obtained from the trial. Statistical significance was evaluated using two-sided tests with a significance level of *p* <0.05.

#### 2.4.1. Efficacy Analysis

For the primary outcome variable (NK cell activity) and secondary outcome variables (cytokine levels, WBC count, and the FSS), within-group comparisons of pre- and post-intervention changes were conducted using paired *t*-tests. Between-group comparisons of changes at each time point (LM1019 group vs. placebo group) were assessed using a two-sample *t*-test or the Wilcoxon rank sum test, depending on whether normality assumptions were met, as determined by the Shapiro-Wilk test.

#### 2.4.2. Demographic and Baseline Characteristics

Demographic data, including age, height, weight, BMI, and lifestyle factors (e.g., alcohol use, smoking status, exercise habits), were summarized using descriptive statistics (number of participants, mean, standard deviation, minimum, and maximum values). For continuous variables, comparisons between groups were made using a two-sample *t*-test or Wilcoxon rank sum test, depending on normality. For categorical variables, the number and percentage of participants were presented, and comparisons between groups were conducted using the Chi-square test or Fisher’s exact test if more than 20% of cells had expected counts less than 5.

#### 2.4.3. Safety Analysis

Safety assessments included the evaluation of adverse events, serious adverse events, and clinical laboratory test results (hematology, blood chemistry, urine tests). Adverse events were summarized by system organ class and preferred term using World Health Organization (WHO) adverse reaction terminology, with the number of participants, incidence rates, and occurrence counts reported for each group. Statistical comparisons between groups were made using the Chi-square test or Fisher’s exact test. For clinical laboratory tests, within-group comparisons of changes were analyzed using paired *t*-tests, and between-group comparisons were conducted using a two-sample *t*-test or Wilcoxon rank sum test, depending on the normality of the data.

#### 2.4.4. Vital Signs, Non-Efficacy Measurements and Other Evaluations

Changes in vital signs (blood pressure, heart rate, body temperature) and other non-efficacy measurements were analyzed using paired *t*-tests for within-group comparisons, and two-sample *t*-tests or Wilcoxon rank sum tests for between-group comparisons, depending on data normality. Nutritional intake was assessed using two complementary methods. First, at visit 2 (week 0), participants completed a 24 h dietary recall, reporting all foods and beverages consumed in the prior day. Second, over the course of the intervention (visits 2–4), participants were provided with a three-day food diary (including two weekdays and one weekend day), which they completed and returned at the subsequent visit. All dietary records were reviewed by the study coordinator for completeness. Nutrient and energy intake were calculated using the Can-Pro 5.0 (Korean Nutrition Society) software, converting all reported foods into their nutrient components according to the Korean Food Composition Table (https://koreanfood.rda.go.kr/kfi/fct/fctIntro/list?menuId=PS03562 (accessed on 21 June 2025)).

## 3. Results

### 3.1. Study Population

**Figure 2:**
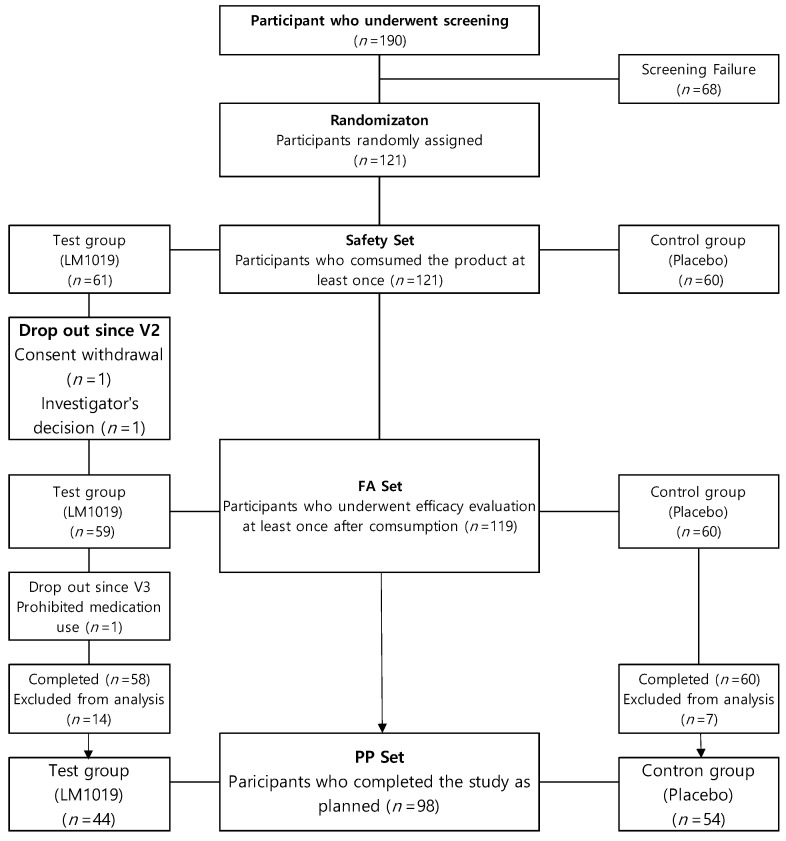
Participant flow chart. The flow chart shows the process of screening, randomization, and analysis set inclusion. Of 190 screened participants, 121 were randomized (test group: LM1019, *n*= 61; control group: placebo, *n* = 60). After dropouts and exclusions, 98 participants completed the study as planned and were included in the PP set (test group: *n* = 44; control group: *n* = 54).

Efficacy was assessed in the per-protocol (PP) and full-analysis (FA) sets (Figure 2). Of 121 randomized subjects who consumed the study product at least once (treatment *n* = 61; control *n* = 60), 119 completed ≥1 efficacy evaluation without major eligibility violations (FA set: treatment *n* = 59; control *n* = 60), with two early withdrawals being excluded. The PP set comprised 98 FA-set participants (treatment *n* = 44; control *n* = 54) who completed the trial without major deviations. Twenty-one subjects were excluded for protocol violations—early discontinuation (*n* = 1), compliance <80% (*n* = 4) or >115% (*n* = 7), alcohol intake change >50 g (*n* = 2), weight change >5% (*n* = 3), abnormal urine results (*n* = 3), or prohibited medication use (*n* = 1).

### 3.2. Baseline Characteristics of the Participants

Baseline characteristics (Table 1) were similar between the LM1019 (*n* = 44) and placebo (*n* = 54) groups. The proportion of females (75.0% vs. 81.5%), mean age (43.98 ± 10.52 vs. 43.76 ± 10.32 years), and BMI (22.67 ± 2.59 vs. 22.18 ± 3.02 kg/m^2^) did not differ (all *p* <0.20). Systolic (116.7 ± 11.0 vs. 112.9 ± 12.2 mmHg) and diastolic (69.6 ± 10.2 vs. 66.8 ± 9.1 mmHg) blood pressures were comparable. Lifestyle factors—including alcohol use (54.6% vs. 51.9%), smoking (non-smokers 97.7% vs. 94.4%), exercise frequency, and Perceived Stress Scale scores (26.20 ± 3.77 vs. 25.52 ± 3.83; *p* = 0.276)—showed no significant differences. Family history of immune disease, comorbidities, surgical history, and medication use were likewise balanced (all *p* < 0.05). COVID-19 vaccination rates were 100% versus 90.7% (*p* = 0.0626).

### 3.3. Safety Assessment

The safety analysis included all 121 randomized participants who consumed the study product at least once (LM1019 *n* = 61; placebo *n* = 60) (Table 2). In the LM1019 group, eight participants (13.1%) experienced mild adverse events and, in the placebo group, eight participants (13.3%) reported events (eleven events total). Respiratory disorders were most common; all were transient, resolved without intervention, judged unrelated to the product, and no serious events occurred.

Laboratory assessments at baseline and week 8 showed that hematological parameters remained within normal clinical ranges. Hemoglobin increased slightly in LM1019 recipients (+0.10 ± 0.69 g/dL) versus a decrease in placebo (−0.18 ± 0.66 g/dL; between-group *p* = 0.0265), and hematocrit followed a similar trend (*p* = 0.0180). Red blood cell counts, platelets, and differential WBCs were stable. In blood biochemistry, AST, HDL-C, and triglycerides exhibited statistically significant intergroup differences (all *p* <0.05) but remained within reference limits. Other markers—including total cholesterol, LDL-C, glucose, hs-CRP, and ESR—showed no notable changes.

Urinalysis detected a small proteinuria difference at week 8 (*p* = 0.0248), which was deemed clinically insignificant. Vital signs and body weight were stable, with only a modest within-group reduction in systolic blood pressure in the LM1019 arm (118.1 → 114.1 mmHg; *p* = 0.0101) without intergroup significance. Overall, LM1019 was well tolerated, with no clinically meaningful safety concerns.

### 3.4. Primary and Secondary Outcomes

NK cell activity, the study’s primary outcome, increased significantly from baseline to week 8 in both LM1019 and placebo groups at all E:T ratios (50:1, 25:1, 12.5:1; all within-group *p* <0.0001), with no significant between-group difference at 50:1 (*p* = 0.9289), 25:1 (*p* = 0.4444), or 12.5:1 (*p* = 0.1511) (Table 3). Secondary outcomes—including serum cytokines (IL-1β, IL-2, IL-6, IL-12, IFN-γ, TNF-α), WBC count, URTI incidence, and Fatigue Severity Scale scores—showed no significant differences between groups at week 8. Within the placebo arm only, IL-6 (*p* = 0.0131) and TNF-α (*p* = 0.0029) declined significantly; cytokine levels in the LM1019 group remained unchanged. WBC counts were stable in both arms (*p* = 0.8513 vs. *p* = 0.1625), URTI cases were few and similar (one vs. two; *p* = 1.0000), and FSS scores improved significantly in both groups (both *p* <0.0001) without intergroup difference (*p* = 0.3952).

### 3.5. Nutritional Intake Analysis

An 8-week dietary assessment (Table 4) showed significant increases in energy intake for both the LM1019 and placebo groups (*p* = 0.0219 and *p* = 0.0266, respectively). The placebo group had an additionally increased intake of protein (*p* = 0.0043), fat (*p* = 0.0323), ash (*p* = 0.0273), vitamin A (*p* = 0.0004), vitamin B6 (*p* = 0.0057), vitamin D (*p* = 0.0020), vitamin E (*p* = 0.0188), beta-carotene (*p* = 0.0010), phosphate (*p* = 0.0043), potassium (*p* = 0.0039), iron (*p* = 0.0085), magnesium (*p* = 0.0060), and zinc (*p* = 0.0014). In the LM1019 group, only energy (*p* = 0.0219) and carbohydrate (*p* = 0.0293) intakes increased significantly, with no other nutrients changing.

### 3.6. Subgroup Analysis

Subgroup analyses were performed post hoc to explore whether baseline markers—specifically age, WBC count, and LDL cholesterol—were associated with differential LM1019 responses in NK cell activity. Each factor was examined independently, and then combined, to identify participant subsets showing greater improvements versus placebo. These exploratory analyses are intended to generate hypotheses for refining future trial designs rather than to confirm efficacy in specific populations.

#### 3.6.1. Age Group

**Figure 3:**
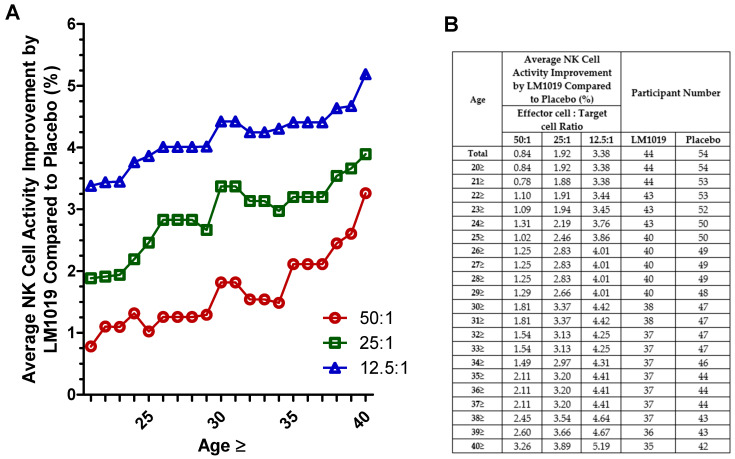
Age-stratified NK cell activity improvement by LM1019 compared with placebo. (**A**) Graph depicting age-stratified NK cell activity improvements by LM1019 compared with placebo at three effector-to-target ratios (50:1, 25:1, and 12.5:1). The x-axis indicates the minimum age in each subgroup (e.g., “25” corresponds to participants aged 25 years and above), and the y-axis shows the relative increase in NK cell activity (%) for the LM1019 group versus the placebo group. (**B**) Accompanying table listing the same age thresholds, the average improvement in NK cell activity at each ratio, and the number of participants in the LM1019 and placebo groups. The table provides a numerical summary of the data displayed in panel (**A**).

In this post-hoc analysis, participants were stratified by age to assess NK cell activity improvements at E:T ratios of 50:1, 25:1, and 12.5:1. As shown in Figure 3A, subgroups aged ≥ 40 years exhibited consistently greater mean increases in NK activity compared with placebo across all ratios, whereas younger subgroups did not demonstrate such improvements. The accompanying tabular data in Figure 3B summarize the average NK cell activity gains and sample sizes at each age cutoff, underscoring the observation that post-hoc analysis identifies older adults as the cohort most responsive to LM1019.

#### 3.6.2. Baseline WBC Count

**Figure 4:**
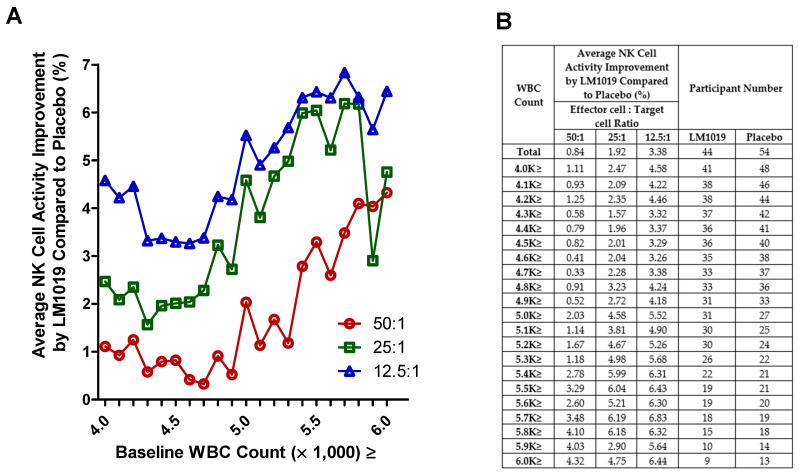
WBC-stratified NK cell activity improvement by LM1019 compared with placebo. (**A**) Post-hoc stratified analysis of NK cell activity categorized by baseline WBC counts. The x-axis denotes the minimum WBC threshold (×1000 cells/μL) retained in each subgroup (e.g., “4.0 K≥” excludes participants whose baseline WBC was below 4.0 K), while the y-axis displays the percentage improvement in NK cell activity for the LM1019 group compared with placebo at three effector-to-target ratios (50:1, 25:1, 12.5:1). (**B**) A tabular summary of these subgroup definitions, listing the average NK cell activity improvement at each WBC threshold and the number of participants in both the LM1019 and placebo groups.

In this post-hoc analysis, baseline WBC counts were stratified to assess their impact on NK cell activity at E:T ratios of 50:1, 25:1, and 12.5:1. As depicted in Figure 4A, progressively excluding participants below the increasing WBC thresholds revealed that those with counts ≥ 5.0 K cells/μL showed larger and more consistent improvements in NK activity versus placebo. The numerical details in Figure 4B confirm that subgroups above this threshold achieved greater mean gains with adequate sample sizes. Participants with lower WBC counts exhibited minimal or inconsistent changes, suggesting that moderate baseline immune competence may enhance responsiveness to LM1019.

#### 3.6.3. Baseline LDL

**Figure 5:**
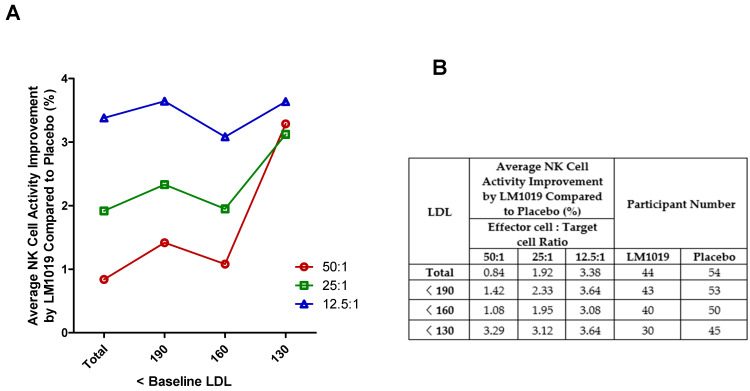
LDL-stratified NK cell activity improvement by LM1019 compared with placebo. (**A**) Graph showing the average NK cell activity improvement (%) by LM1019 compared with placebo across different baseline LDL cholesterol levels (mg/dL) in the PP set. The x-axis represents progressively stricter LDL thresholds (<190, <160, <130 mg/dL), excluding participants with higher LDL levels stepwise. The y-axis displays the relative improvement in NK cell activity at effector-to-target (E:T) ratios of 50:1, 25:1, and 12.5:1. (**B**) Table summarizing the numerical data corresponding to panel (**A**). This table presents the average NK cell activity improvement at each LDL threshold, stratified by E:T ratios (50:1, 25:1, and 12.5:1) along with the number of participants analyzed in the LM1019 and placebo groups.

In this post-hoc analysis, participants were stratified by baseline LDL cholesterol into <190, <160, and <130 mg/dL to evaluate NK cell activity at E:T ratios of 50:1, 25:1, and 12.5:1 (Figure 5A,B). Across the entire PP set (*n* = 44 LM1019; *n* = 54 placebo), LM1019 induced modest mean improvements of 0.84%, 1.92%, and 3.38% at 50:1, 25:1, and 12.5:1, respectively. Excluding those with LDL ≥ 190 mg/dL (*n* = 43 vs. 53), the <190 mg/dL subgroup showed enhanced gains of 1.42%, 2.33%, and 3.64%. Further restricting to LDL < 160 mg/dL (*n* = 40 vs. 50) yielded mean improvements of 1.08%, 1.95%, and 3.08%. Most notably, participants with LDL < 130 mg/dL (*n* = 30 vs. 45) experienced the largest benefits—3.29%, 3.12%, and 3.64% increases at 50:1, 25:1, and 12.5:1, respectively—demonstrating a clear trend toward greater LM1019 efficacy in those with lower LDL levels. These data (Figure 5A) and the tabulated values (Figure 5B) support the hypothesis that tighter LDL control may potentiate the immunostimulatory effects of LM1019.

#### 3.6.4. Combined Subgroup Analysis

In this post-hoc analysis, participants meeting all three criteria—age ≥ 40 years, baseline WBC ≥ 5.0 K cells/μL, and LDL < 130 mg/dL—showed the most pronounced NK cell activity gains after 8 weeks (Table 5). At the 50:1 E:T ratio, LM1019 recipients (*n* = 18) increased from 50.72 ± 12.33% to 60.98 ± 11.13% (*p* = 0.0004) versus 55.58 ± 10.31% to 57.23 ± 8.87% in placebo (*n* = 17; *p* = 0.5472), with a between-group *p* = 0.0210. At 25:1, LM1019 rose from 38.59 ± 13.11% to 48.76 ± 14.15% (*p* = 0.0032) versus 44.25 ± 11.21% to 44.55 ± 10.79% (*p* = 0.9276; between-group *p* = 0.0297). At 12.5:1, LM1019 rose from 21.07 ± 9.57% to 29.04 ± 11.57% (*p* = 0.0002) compared with 26.24 ± 8.78% to 25.61 ± 7.98% in placebo (*p* = 0.8093; between-group *p* = 0.0080). These results indicate that combining age, WBC, and LDL criteria identifies a subgroup in which LM1019 elicits statistically significant improvements in NK cell cytotoxicity.

## 4. Discussion

### 4.1. Main Findings

LM1019 supplementation selectively enhanced NK cell cytotoxicity in a targeted subgroup (aged ≥ 40 years with WBC ≥ 5.0 × 10^3^/µL and LDL < 130 mg/dL) without elevating systemic cytokines. This immune modulation pattern—boosting innate antiviral responses without systemic inflammation—suggests regulated innate immune engagement. A plausible mechanism involves gut dendritic cells (DCs), known to stimulate NK cells by secreting cytokines like IL-12 and IL-15. Lactobacilli probiotics, including *Lacticaseibacillus rhamnosus* GG, have demonstrated NK activation via DC-mediated pathways [44]. A clinical trial in elderly adults showed *L. rhamnosus* GG combined with prebiotic fiber significantly increased NK cell activity and decreased pro-inflammatory markers (e.g., IL-6), alongside cholesterol reduction [38]. Similarly, trials using *L. casei* Shirota have reported enhanced NK cytotoxicity in individuals with lower baseline NK activity [45], aligning with our findings in participants ≥ 40 years experiencing immunosenescence.

Underlying this is the IL-10/IL-12 regulatory balance induced by *L. rhamnosus* strains in antigen-presenting cells, promoting moderate IL-12-driven NK and T-cell activity alongside robust IL-10 expression to control inflammation [45,46,47]. In our context, LM1019 likely activates gut DCs or macrophages locally, triggering NK-activating factors without systemic inflammatory cytokine overflow. However, as we did not directly measure these mediators, this mechanism remains speculative. LM1019 seems to activate innate immunity in a controlled manner, analogous to other *L. rhamnosus* strains that enhance NK function while preserving immunological homeostasis.

### 4.2. Study Limitations and Considerations

Despite promising findings, several limitations should be noted. First, NK cell enhancement was confined to a subgroup defined by age, WBC, and LDL criteria, limiting generalizability. Baseline immune status appears influential; middle-aged or older adults with moderate LDL and reduced chronic inflammation may benefit most due to room for immune improvement. Younger participants with already robust NK activity might experience a ceiling effect, reducing detectable benefits. Consequently, caution is needed when extrapolating results to populations outside these criteria. Further studies should stratify participants by immune/metabolic markers to clarify LM1019’s effectiveness.

Second, we measured systemic cytokines without examining local immune responses or transient fluctuations. Absence of systemic cytokine elevation indicates safety but does not exclude local gut mucosal activity. Proposed DC-NK cell mechanisms remain inferred from analogous studies; direct measurement of cytokines such as IL-12 or IL-10 was not conducted. Transient or localized cytokine responses may have occurred undetected in peripheral blood.

Third, the supplementation duration was relatively short, focusing solely on surrogate markers rather than clinical endpoints. Long-term sustainability of NK enhancements and translation into clinical benefits (e.g., infection resistance) remain unclear. Additionally, strain-specific biological differences were not explored due to the study scope. Though LM1019 appears similar to certain *L. rhamnosus* strains in balanced NK activation, probiotic effects can be strain dependent. Future comparative studies could clarify LM1019’s specific immune-regulatory positioning.

Finally, dietary assessments revealed significant dietary modifications in the placebo arm, including increased intake of protein, fat, vitamins A, D, E, B6, zinc, iron, magnesium, and phosphate, nutrients known for immune modulation [48,49]. Protein is essential for NK and lymphocyte proliferation and function [50]. Zinc supplementation directly enhances NK cell cytotoxicity [51,52]. Vitamin D suppresses pro-inflammatory cytokines (IL-6, TNF-α) and promotes antimicrobial peptide production [51,52]. Vitamin E boosts T-cell responses, NK function, and reduces inflammation [53,54]. Vitamin B6 and iron are essential for lymphocyte proliferation and antibody production [55,56]. Such dietary improvements likely increased baseline immune function in the placebo group, confounding LM1019 efficacy assessment.

### 4.3. Future Research Directions

Future studies should integrate mechanistic and clinical investigations. Detailed immunological studies could confirm LM1019’s interaction with antigen-presenting and NK cells, measuring local cytokine responses directly. Sampling gut mucosa could reveal localized immune responses. Clinical benefits should be evaluated by assessing infection rates or vaccine responses in LM1019-supplemented groups versus placebo. Larger, diverse trials stratified by age, baseline NK function, and metabolic biomarkers will identify ideal candidates for LM1019. Synbiotic approaches combining LM1019 with prebiotic fibers could also broaden effectiveness. Long-term studies must ensure sustained immune balance and safety. These combined efforts will comprehensively characterize LM1019’s potential, connecting mechanistic insights with clinical outcomes, supporting targeted immune supplementation strategies.

## 5. Conclusions

In this randomized, placebo-controlled clinical trial, *Lacticaseibacillus rhamnosus* LM1019 did not significantly enhance NK cell activity across the entire cohort. However, post-hoc analyses identified a responsive subgroup—adults aged ≥ 40 years with preserved immune profiles (WBC ≥ 5.0 × 10^3^/μL) and optimal lipid levels (LDL < 130 mg/dL)—in whom LM1019 significantly improved NK cytotoxicity without elevating systemic cytokines. These findings suggest phenotype-specific immunomodulatory potential, particularly in older adults with modifiable immunity.

Importantly, LM1019 was well-tolerated with no significant safety concerns reported. Observed improvements occurred in the absence of systemic inflammatory cytokine elevations, supporting a plausible mucosal mechanism of action. However, dietary variability—especially spontaneous nutritional improvements in the placebo group—likely attenuated between-group differences and highlights the need for tighter dietary control in future trials.

To validate these findings, future studies should include stratified participant selection, longer intervention durations, and direct mechanistic investigations (e.g., mucosal cytokine responses, DC-NK cell crosstalk). Such efforts will be essential to fully define the clinical relevance and mechanistic basis of LM1019’s immune-supportive effects.

## Figures and Tables

**Table 1:** Baseline characteristics of the participants (PP Set).

	LM1019 (*n* = 44)	Placebo (*n* = 54)	*p*-Value
Sex			0.4367(C)
Male	11 (25.00%)	10 (18.52%)	
Female	33 (75.00%)	44 (81.48%)	
Age (years)	43.98 ± 10.52	43.76 ± 10.32	0.9181(T)
BMI (kg/m^2^)	22.67 ± 2.59	22.18 ± 3.02	0.2099(W)
Systolic blood pressure (mmHg)	116.73 ± 10.96	112.87 ± 12.16	0.1061(T)
Diastolic blood pressure (mmHg)	69.55 ± 10.22	66.80 ± 9.12	0.1632(T)
Family history of immune diseases	2 (4.55%)	1 (1.85%)	0.5860(F)
COVID-19 vaccination	44 (100%)	49 (90.74%)	0.0626(F)
Alcohol consumers	24 (54.55%)	28 (51.85%)	0.7904(C)
Tobacco users			
Non-smokers	43 (97.73%)	51 (94.44%)	0.4126(F)
Former smokers (>1 year)	0 (0%)	2 (3.70%)	
Former smokers (<1 year)	0 (0%)	1 (1.85%)	
Smokers	1 (2.27%)	0 (0%)	
Exercise			0.6389(F)
No exercise	8 (18.18%)	8 (14.81%)	
1–2 times/week	19 (43.18%)	27 (50.00%)	
3–4 times/week	11 (25.00%)	10 (18.52%)	
5–6 times/week	2 (4.55%)	6 (11.11%)	
7 times/week	4 (9.09%)	3 (5.56%)	
PSS (point)	26.20 ± 3.77	25.52 ± 3.83	0.2756(W)

Values are presented as *n* (%) for categorical variables and mean ± standard deviation (SD) for continuous variables. *p*-values compare the two groups at baseline. (C) Chi-square test, (F) Fisher’s exact test, (T) two-sample *t*-test, (W) Wilcoxon rank sum test.

**Table 2:** Changes in safety parameters.

Variables	LM1019 (*n* = 61)				Placebo (*n* = 60)				Between Groups ^(2)^
	Baseline		8 Weeks	Within Group ^(1)^	Baseline	8 Weeks		Within Group ^(1)^	
Vital Parameters									
Weight (kg)	61.60 ± 11.02		61.74 ± 10.73	0.3230	59.52 ± 10.95	59.76 ± 11.41		0.1806	0.8879(T)
Systolic blood pressure (mmHg)	118.07 ± 11.22		114.14 ± 12.90	**0.0101**	113.02 ± 12.16	111.32 ± 9.59		0.2037	0.3009(T)
Diastolic blood pressure (mmHg)	69.93 ± 9.89		67.78 ± 10.47	0.0548	67.03 ± 8.86	66.40 ± 8.93		0.5343	0.3651(T)
Body temperature (°C)	36.50 ± 0.12		36.50 ± 0.10	0.8253	36.51 ± 0.11	36.49 ± 0.10		0.1889	0.3646(W)
Heart rate (bpm)	77.98 ± 10.93		76.53 ± 9.74	0.1806	79.80 ± 10.09	77.73 ± 8.93		0.1097	0.7114(T)
Hematology Test									
RBC (10^6^/μL)	4.49 ± 0.43		4.51 ± 0.50	0.5923	4.42 ± 0.37	4.37 ± 0.39		0.0688	0.0636(W)
Hemoglobin (g/dL)	13.62 ± 1.33		13.72 ± 1.44	0.2637	13.37 ± 1.19	13.19 ± 1.29		0.0423	**0.0265(T)**
Hematocrit (%)	41.40 ± 3.47		41.54 ± 4.02	0.6397	40.84 ± 3.34	39.99 ± 3.64		**0.0063**	**0.0180(T)**
Platelet (10^3^/μL)	266.26 ± 64.73		257.88 ± 55.82	0.2342	260.57 ± 54.27	265.42 ± 54.19		0.1880	0.0759(T)
Seg. Neutrophil (%)	52.36 ± 8.84		53.27 ± 7.94	0.2286	50.96 ± 8.51	52.56 ± 7.39		0.1607	0.9464(W)
Lymphocyte (%)	37.55 ± 7.82		36.75 ± 7.37	0.2283	38.27 ± 7.40	37.23 ± 7.23		0.2793	0.7961(W)
Monocyte (%)	7.03 ± 1.90		6.74 ± 1.69	0.0679	6.95 ± 1.83	6.82 ± 1.80		0.4314	0.5234(T)
Eosinophil (%)	2.28 ± 1.81		2.46 ± 1.66	0.3532	3.07 ± 3.11	2.65 ± 2.29		0.0722	0.0632(W)
Basophil (%)	0.78 ± 0.36		0.77 ± 0.35	0.9576	0.75 ± 0.29	0.73 ± 0.34		0.5776	0.7056(T)
Blood Biochemical Test									
AST (U/L)	21.77 ± 5.30		21.02 ± 4.91	0.0770	21.95 ± 6.12	23.08 ± 8.52		0.1756	0.0702(W)
ALT (U/L)	17.03 ± 8.28		17.55 ± 8.66	0.9015	17.47 ± 10.83	20.02 ± 15.23		**0.0297**	0.2148(W)
γ-GTP (U/L)	21.23 ± 9.93		22.50 ± 13.31	0.2242	19.48 ± 9.61	21.10 ± 12.46		**0.0153**	0.8901(W)
Glucose	90.20 ± 8.87		90.81 ± 8.70	0.7760	88.23 ± 6.35	88.47 ± 7.82		0.7442	0.6778(W)
Total cholesterol (mg/dL)	198.66 ± 30.75		194.07 ± 30.53	0.1941	191.92 ± 27.76	196.63 ± 32.98		0.1342	0.0743(W)
HDL cholesterol (mg/dL)	59.52 ± 13.76		58.21 ± 13.27	0.4662	61.25 ± 11.34	63.43 ± 12.06		**0.0352**	**0.0433(T)**
LDL cholesterol (mg/dL)	117.41 ± 26.44		112.93 ± 26.33	0.0651	110.97 ± 27.26	113.55 ± 31.34		0.2918	0.1536(W)
Triglyceride (mg/dL)	93.57 ± 56.79		104.17 ± 69.47	**0.0196**	83.77 ± 45.69	82.05 ± 42.98		0.7272	0.1394(W)
hs-CRP (mg/L)	0.58 ± 0.86		0.69 ± 1.29	0.6049	0.58 ± 0.96	0.52 ± 0.55		0.6027	0.1705(W)
ESR (mm/h)	5.97 ± 7.31		6.67 ± 7.48	0.4067	6.03 ± 7.83	6.62 ± 7.25		0.2863	0.4636(W)
Urinalysis									
Protein	Normal	→	Normal (*n* = 41)	0.3173 (M)	Normal	→	Normal (*n*= 45)	0.7055 (M)	**0.0248(F)**
	Normal	→	Abnormal (*n* = 10)		Normal	→	Abnormal (*n* = 4)		
	Abnormal	→	Normal (*n* = 6)		Abnormal	→	Normal (*n* = 3)		
	Abnormal	→	Abnormal (*n* = 1)		Abnormal	→	Abnormal (*n* = 8)		
Glucose	Normal	→	Normal (*n* = 57)	-	Normal	→	Normal (*n* = 59)	-	0.7436(F)
	Normal	→	Abnormal (*n* = 0)		Normal	→	Abnormal (*n* = 1)		
	Abnormal	→	Normal (*n* = 1)		Abnormal	→	Normal (*n* = 0)		
	Abnormal	→	Abnormal (*n* = 0)		Abnormal	→	Abnormal (*n* = 0)		
Ketone	Normal	→	Normal (*n* = 57)	-	Normal	→	Normal (*n* = 58)	-	1.0000(F)
	Normal	→	Abnormal (*n* = 0)		Normal	→	Abnormal (*n* = 0)		
	Abnormal	→	Normal (*n* = 1)		Abnormal	→	Normal (*n* = 2)		
	Abnormal	→	Abnormal (*n* = 0)		Abnormal	→	Abnormal (*n* = 0)		
Erythrocytes	Normal	→	Normal (*n* = 38)	0.6171 (M)	Normal	→	Normal (*n* = 49)	0.4795 (M)	0.1719(F)
	Normal	→	Abnormal (*n* = 7)		Normal	→	Abnormal (*n* = 5)		
	Abnormal	→	Normal (*n* = 9)		Abnormal	→	Normal (*n* = 3)		
	Abnormal	→	Abnormal (*n* = 4)		Abnormal	→	Abnormal (*n* = 3)		
Leukocytes	Normal	→	Normal (*n* = 37)	-	Normal	→	Normal (*n* = 45)	-	-
	Normal	→	Abnormal (*n* = 7)		Normal	→	Abnormal (*n* = 7)		
	Abnormal	→	Normal (*n* = 5)		Abnormal	→	Normal (*n* = 5)		
	Abnormal	→	Abnormal (*n* = 9)		Abnormal	→	Abnormal (*n* = 3)		
Nitrite	Normal	→	Normal (*n* = 54)	0.3173 (M)	Normal	→	Normal (*n* = 58)	-	0.0566(F)
	Normal	→	Abnormal (*n* = 0)		Normal	→	Abnormal (*n* = 2)		
	Abnormal	→	Normal (*n* = 1)		Abnormal	→	Normal (*n* = 0)		
	Abnormal	→	Abnormal (*n* = 3)		Abnormal	→	Abnormal (*n* = 0)		

Values are presented as mean ± standard deviation (SD) for continuous variables. Within-group *p*-values ^(1)^ compare baseline and 8-week measurements within each group. Unless otherwise indicated, a paired *t*-test was performed for within-group comparisons. Between-group *p*-values ^(2)^ compare the changes from baseline to 8 weeks between the LM1019 and placebo groups. Statistical tests used are indicated by letters in parentheses: (T) Paired *t*-test, (W) Wilcoxon signed-rank test, (M) McNemar’s test, and (F) Fisher’s exact test. Abbreviations: RBC: Red blood cells, AST: Aspartate aminotransferase, ALT: Alanine aminotransferase, γ-GTP: Gamma-glutamyl transferase, HDL-C: High-density lipoprotein cholesterol, LDL-C: Low-density lipoprotein cholesterol, hs-CRP: High-sensitivity C-reactive protein, ESR: Erythrocyte sedimentation rate, bpm: Beats per minute, N: Number of participants. For urinalysis results, values indicate the number of participants (n) whose test results changed from baseline to week 8: Normal → Normal: Participants with normal results at both baseline and week 8, Normal → Abnormal: Participants whose results changed from normal at baseline to abnormal at week 8, Abnormal → Normal: Participants whose results changed from abnormal at baseline to normal at week 8, Abnormal → Abnormal: Participants with abnormal results at both baseline and week 8.

**Table 3:** Changes in primary and secondary outcomes from baseline to week 8.

Variables	LM1019 (*n* = 44)			Placebo (*n* = 54)			Between Groups ^(2)^
	Baseline	8 Weeks	Within Group ^(1)^	Baseline	8 Weeks	Within Group ^(1)^	
NK cell activity (%)							
E:T = 50:1	51.59 ± 10.84	60.63 ± 9.98	**<0.0001**	52.00 ± 12.28	60.20 ± 9.51	**<0.0001**	0.9289(W)
E:T = 25:1	38.86 ± 11.48	48.53 ± 11.20	**<0.0001**	40.76 ± 13.29	48.51 ± 10.54	**<0.0001**	0.4444(T)
E:T = 12.5:1	20.72 ± 8.48	28.28 ± 9.79	**<0.0001**	23.99 ± 9.88	28.16 ± 7.82	**0.0030**	0.1511(W)
Cytokines (pg/mL)							
IL-1β	1.57 ± 1.00	1.38 ± 0.78	0.0626	2.14 ± 3.49	1.80 ± 2.27	0.0671	0.3933(W)
IL-2	0.75 ± 0.35	0.73 ± 0.33	0.7780	1.14 ± 2.54	0.98 ± 1.52	0.2534	0.4092(W)
IL-6	9.06 ± 15.66	7.53 ± 12.64	0.1063	11.64 ± 18.10	9.59 ± 14.55	**0.0131**	0.2274(W)
IL-12	3.95 ± 10.51	2.15 ± 2.44	0.1984	2.85 ± 6.79	2.70 ± 5.14	0.6468	0.6630(W)
IFN-γ	6.73 ± 8.24	6.10 ± 6.18	0.3315	6.65 ± 6.93	6.84 ± 7.64	0.7833	0.1923(W)
TNF-α	9.81 ± 4.87	8.84 ± 4.68	0.0757	10.39 ± 5.23	8.96 ± 4.75	**0.0029**	0.3914(W)
WBC count (10^3^ cells/μL)	5.33 ± 0.87	5.30 ± 1.20	0.8513	5.20 ± 1.00	4.98 ± 1.19	0.1625	0.5320(W)
URTI (case)	-	1	-	-	2	-	1.0000(F)
FSS (point)	4.88 ± 1.16	3.68 ± 1.00	**<0.0001**	4.97 ± 0.92	3.82 ± 0.96	**<0.0001**	0.3952(W)

Values are presented as mean ± standard deviation (SD) for continuous variables. Within-group *p*-values ^(1)^ compare baseline and 8-week measurements within each group. Between-group *p*-values ^(2)^ compare the changes from baseline to 8 weeks between the LM1019 and placebo groups. Statistical tests used are indicated by letters in parentheses: (T): Paired *t*-test, (W): Wilcoxon signed-rank test, and (F): Fisher’s exact test. NK cell cytotoxic activity measured at effector-to-target (E:T) ratios of 50:1, 25:1, and 12.5:1. WBC count: White blood cell count.

**Table 4:** Changes in nutrient intake from baseline to week 8.

Variables	LM1019 (*n* = 44)			Placebo (*n* = 54)			Between Groups ^(2)^
	Baseline	8 Weeks	Within Group ^(1)^	Baseline	8 Weeks	Within Group ^(1)^	
Nutritional Analysis							
Energy intake (kcal)	1474.50 ± 596.60	1679.81 ± 552.54	**0.0219**	1463.65 ± 485.52	1607.18 ± 335.96	**0.0266**	0.5559(T)
Carbohydrate (g)	195.80 ± 66.38	220.25 ± 70.64	**0.0293**	202.59 ± 66.21	212.86 ± 52.22	0.2080	0.2869(T)
Protein (g)	63.31 ± 37.65	67.68 ± 26.	0.4486	58.52 ± 21.44	66.74 ± 16.37	**0.0043**	0.8723(W)
Fat (g)	47.03 ± 30.14	56.64 ± 29.36	0.0598	44.28 ± 25.60	52.29 ± 18.83	**0.0323**	0.7915(T)
Dietary fiber (g)	15.27 ± 7.76	16.79 ± 6.56	0.1463	14.87 ± 6.38	16.67 ± 5.85	0.0592	0.8949(W)
Ash (g)	14.30 ± 6.23	14.93 ± 5.23	0.4489	12.87 ± 4.73	14.24 ± 3.31	**0.0273**	0.4637(T)
Vitamin A (μg)	462.18 ± 317.05	522.97 ± 271.30	0.2678	392.02 ± 212.08	548.60 ± 232.97	**0.0004**	0.3261(W)
Vitamin B6 (mg)	0.44 ± 0.42	0.54 ± 0.38	0.2628	0.36 ± 0.35	0.55 ± 0.29	**0.0057**	0.2921(W)
Vitamin B12 (μg)	3.61 ± 4.41	4.01 ± 2.84	0.5533	3.77 ± 6.04	4.51 ± 6.90	0.0790	0.6971(W)
Vitamin C (mg)	46.37 ± 64.52	58.01 ± 38.74	0.2914	49.78 ± 53.93	58.11 ± 39.27	0.3519	0.4554(W)
Vitamin D (μg)	1.68 ± 2.42	1.69 ± 2.03	0.9798	0.88 ± 1.05	1.50 ± 1.21	**0.0020**	0.1973(W)
Vitamin E (mg)	8.89 ± 5.94	9.71 ± 4.13	0.3727	8.22 ± 4.39	9.89 ± 4.14	**0.0188**	0.4511(W)
Beta-carotene (μg)	1748.81 ± 1198.00	2131.89 ± 1044.68	0.0754	1590.54 ± 987.92	2340.80 ± 1358.65	**0.0010**	0.4862(W)
Calcium (mg)	358.90 ± 181.35	420.14 ± 238.54	0.1431	359.31 ± 265.86	387.87 ± 144.63	0.4148	0.6605(W)
Phosphate (mg)	900.40 ± 478.83	958.97 ± 371.74	0.4296	828.68 ± 336.18	950.96 ± 222.26	**0.0043**	0.5557(W)
Sodium (mg)	3115.57 ± 1487.38	3206.38 ± 1233.22	0.6714	2805.14 ± 1215.31	2921.10 ± 827.99	0.4827	0.9244(T)
Potassium (mg)	1903.27 ± 910.67	2080.52 ± 689.16	0.1828	1811.26 ± 656.52	2094.46 ± 561.02	**0.0039**	0.7615(W)
Iron (mg)	10.82 ± 6.53	10.18 ± 4.30	0.6057	8.70 ± 3.86	10.25 ± 3.11	**0.0085**	0.2954(W)
Magnesium (mg)	168.22 ± 82.75	178.78 ± 73.73	0.4373	158.70 ± 70.84	188.65 ± 67.23	**0.0060**	0.2512(T)
Zinc (mg)	6.61 ± 3.85	6.94 ± 2.80	0.5407	5.72 ± 2.49	7.15 ± 2.58	**0.0014**	0.1924(W)

Values are presented as mean ± standard deviation (SD). Within-group *p*-values ^(1)^ compare baseline and 8-week measurements within each group. Between-group *p*-values ^(2)^ compare the changes from baseline to 8 weeks between the LM1019 and placebo groups. Statistical tests used are indicated by letters in parentheses: (T): Paired *t*-test, and (W): Wilcoxon signed-rank test.

**Table 5:** Summary of subgroup analyses for changes in NK cell activity.

Subgroup Analyses	NK Cell Activity	Group	*n*	Baseline	8 Weeks	Within Group ^(1)^	Between Groups ^(2)^
Age ≥ 40	E:T = 50:1	LM1019	35	51.69 ± 10.45	61.25 ± 10.59	<0.0001	0.2540(W)
		Placebo	42	53.97 ± 11.60	60.27 ± 9.92	0.0008	
	E:T = 25:1	LM1019	35	39.26 ± 11.53	49.27 ± 11.90	<0.0001	0.2139(W)
		Placebo	42	42.96 ± 12.26	49.08 ± 10.83	0.0024	
	E:T = 12.5:1	LM1019	35	20.92 ± 8.85	26.17 ± 9.21	<0.0001	**0.0439(W)**
		Placebo	42	28.79 ± 10.40	28.85 ± 7.87	0.0902	
WBC ≥ 5.0 k	E:T = 50:1	LM1019	31	51.29 ± 11.51	59.85 ± 9.42	<0.0001	0.4978(T)
		Placebo	27	51.59 ± 13.51	58.11 ± 9.86	0.0118	
	E:T = 25:1	LM1019	31	38.18 ± 11.85	47.91 ± 11.88	<0.0001	0.1694(T)
		Placebo	27	40.67 ± 14.25	45.81 ± 10.78	0.0670	
	E:T = 12.5:1	LM1019	31	19.85 ± 8.53	27.86 ± 9.94	<0.0001	**0.0215(T)**
		Placebo	27	23.50 ± 9.97	26.00 ± 7.70	0.2209	
LDL < 130 mg/dL	E:T = 50:1	LM1019	30	51.65 ± 12.03	61.28 ± 10.89	<0.0001	0.1799(T)
		Placebo	45	53.83 ± 10.70	60.17 ± 8.87	0.0002	
	E:T = 25:1	LM1019	30	39.22 ± 12.24	48.68 ± 11.85	0.0001	0.2822(T)
		Placebo	45	42.47 ± 12.38	48.81 ± 10.54	0.0015	
	E:T = 12.5:1	LM1019	30	21.70 ± 9.04	28.62 ± 10.44	0.0002	0.1246(W)
		Placebo	45	25.22 ± 9.55	28.50 ± 8.06	0.0335	
Age ≥ 40, WBC ≥ 5.0 k, LDL < 130 mg/dL	E:T = 50:1	LM1019	18	50.72 ± 12.33	60.98 ± 11.13	0.0004	**0.0210(T)**
		Placebo	17	55.58 ± 10.31	57.23 ± 8.87	0.5472	
	E:T = 25:1	LM1019	18	38.59 ± 13.11	48.76 ± 14.15	0.0032	**0.0297(T)**
		Placebo	17	44.25 ± 11.21	44.55 ± 10.79	0.9276	
	E:T = 12.5:1	LM1019	18	21.07 ± 9.57	29.04 ± 11.57	0.0002	**0.0080(T)**
		Placebo	17	26.24 ± 8.78	25.61 ± 7.98	0.8093	

Presented are the changes in NK cell activity at weeks 0 and 8 for subgroups treated with LM1019 or placebo. Subgroups include participants aged ≥ 40, those with baseline WBC counts ≥ 5.0 k, LDL cholesterol < 130 mg/dL, and individuals meeting all three combined criteria. Results cover E:T ratios of 50:1, 25:1, and 12.5:1. Participant number is denoted as “*n*”. ^(1)^ Within-group comparisons used paired *t*-tests unless specified otherwise. ^(2)^ Between-group comparisons utilized appropriate statistical tests (T: two-sample *t*-test, W: Wilcoxon rank-sum test).

## Data Availability

Data are contained within the article.

## References

[1] Goyani P., Christodoulou R., Vassiliou E. (2024). Immunosenescence: Aging and Immune System Decline. Vaccines.

[2] Elyahu Y., Monsonego A. (2021). Thymus involution sets the clock of the aging T-cell landscape: Implications for declined immunity and tissue repair. Ageing Res. Rev..

[3] Qi Q., Liu Y., Cheng Y., Glanville J., Zhang D., Lee J.-Y., Olshen R.A., Weyand C.M., Boyd S.D., Goronzy J.J. (2014). Diversity and clonal selection in the human T-cell repertoire. Proc. Natl. Acad. Sci. USA.

[4] Cardinale A., De Luca C.D., Locatelli F., Velardi E. (2021). Thymic Function and T-Cell Receptor Repertoire Diversity: Implications for Patient Response to Checkpoint Blockade Immunotherapy. Front. Immunol..

[5] Britanova O.V., Putintseva E.V., Shugay M., Merzlyak E.M., Turchaninova M.A., Staroverov D.B., Bolotin D.A., Lukyanov S., Bogdanova E.A., Mamedov I.Z. (2014). Age-related decrease in TCR repertoire diversity measured with deep and normalized sequence profiling. J. Immunol..

[6] Egorov E.S., Kasatskaya S.A., Zubov V.N., Izraelson M., Nakonechnaya T.O., Staroverov D.B., Angius A., Cucca F., Mamedov I.Z., Rosati E. (2018). The Changing Landscape of Naive T Cell Receptor Repertoire with Human Aging. Front. Immunol..

[7] Chang S.T., Chuang Y.F., Li A.H., Fan Y.T., Liao M.R., Chen I.Y., Hung R.W., Yang T.O., Chiu Y.L. (2024). Age-dependent immune profile in healthy individuals: An original study, systematic review and meta-analysis. Immun. Ageing.

[8] Tedeschi V., Paldino G., Kunkl M., Paroli M., Sorrentino R., Tuosto L., Fiorillo M.T. (2022). CD8⁺ T Cell Senescence: Lights and Shadows in Viral Infections, Autoimmune Disorders and Cancer. Int. J. Mol. Sci..

[9] Lin Y., Damjanovic A., Metter E.J., Nguyen H., Truong T., Najarro K., Morris C., Longo D.L., Zhan M., Ferrucci L. (2015). Age-associated telomere attrition of lymphocytes in vivo is coordinated with changes in telomerase activity, composition of lymphocyte subsets and health conditions. Clin. Sci..

[10] Naylor K., Li G., Vallejo A.N., Lee W.-W., Koetz K., Bryl E., Witkowski J., Fulbright J., Weyand C.M., Goronzy J.J. (2005). The influence of age on T cell generation and TCR diversity. J. Immunol..

[11] de Mol J., Kuiper J., Tsiantoulas D., Foks A.C. (2021). The Dynamics of B Cell Aging in Health and Disease. Front. Immunol..

[12] Siegrist C.A., Aspinall R. (2009). B-cell responses to vaccination at the extremes of age. Nat. Rev. Immunol..

[13] Shaw A.C., Joshi S., Greenwood H., Panda A., Lord J.M. (2010). Aging of the innate immune system. Curr. Opin. Immunol..

[14] Puzianowska-Kuźnicka M., Owczarz M., Wieczorowska-Tobis K., Nadrowski P., Chudek J., Ślusarczyk P., Skalska A., Jonas M., Franek E., Mossakowska M. (2016). Interleukin-6 and C-reactive protein, successful aging, and mortality: The PolSenior study. Immun. Ageing.

[15] Ferrucci L., Fabbri E. (2018). Inflammageing: Chronic inflammation in ageing, cardiovascular disease, and frailty. Nat. Rev. Cardiol..

[16] Fulop T., Larbi A., Dupuis G., Le Page A., Frost E.H., Cohen A.A., Witkowski J.M., Franceschi C. (2018). Immunosenescence and inflammaging as two sides of the same coin: Friends or foes?. Front. Immunol..

[17] Weinberger B., Herndler-Brandstetter D., Schwanninger A., Weiskopf D., Grubeck-Loebenstein B. (2008). Biology of immune responses to vaccines in elderly persons. Clin. Infect. Dis..

[18] Goronzy J.J., Weyand C.M. (2012). Immune aging and autoimmunity. Cell. Mol. Life Sci..

[19] Campisi J. (2013). Aging, cellular senescence, and cancer. Annu. Rev. Physiol..

[20] Bauer M.E. (2020). Accelerated immunosenescence in rheumatoid arthritis: Impact on clinical progression. Immun. Ageing.

[21] Tay M.Z., Poh C.M., Rénia L., MacAry P.A., Ng L.F.P. (2020). The trinity of COVID-19: Immunity, inflammation and intervention. Nat. Rev. Immunol..

[22] Mak J.W.Y., Chan F.K.L., Ng S.C. (2020). Probiotics and COVID-19: One size does not fit all. Lancet Gastroenterol. Hepatol..

[23] Chen Y., Klein S.L., Garibaldi B.T., Li H., Wu C., Osevala N.M., Li D., Zhang F., Klein C., Jin F. (2021). Aging in COVID-19: Vulnerability, immunity and intervention. Ageing Res. Rev..

[24] Uddin N., Acter T., Rashid M.H., Chowdhury A.I., Jahan E.A. (2023). Coping with the COVID-19 pandemic by strengthening immunity as a nonpharmaceutical intervention: A major public health challenge. Health Sci. Rep..

[25] Yan F., Polk D.B. (2011). Probiotics and immune health. Curr. Opin. Gastroenterol..

[26] Mazziotta C., Tognon M., Martini F., Torreggiani E., Rotondo J.C. (2023). Probiotics Mechanism of Action on Immune Cells and Beneficial Effects on Human Health. Cells.

[27] Chandrasekaran P., Weiskirchen S., Weiskirchen R. (2024). Effects of Probiotics on Gut Microbiota: An Overview. Int. J. Mol. Sci..

[28] Markowiak-Kopeć P., Śliżewska K. (2020). The Effect of Probiotics on the Production of Short-Chain Fatty Acids by Human Intestinal Microbiome. Nutrients.

[29] Azad M.A.K., Sarker M., Wan D. (2018). Immunomodulatory Effects of Probiotics on Cytokine Profiles. Biomed. Res. Int..

[30] Björkström N.K., Strunz B., Ljunggren H.-G. (2022). Natural killer cells in antiviral immunity. Nat. Rev. Immunol..

[31] Wang R., Jaw J.J., Stutzman N.C., Zou Z., Sun P.D. (2012). Natural killer cell-produced IFN-γ and TNF-α induce target cell cytolysis through up-regulation of ICAM-1. J. Leukoc. Biol..

[32] Mujal A.M., Delconte R.B., Sun J.C. (2021). Natural Killer Cells: From Innate to Adaptive Features. Annu. Rev. Immunol..

[33] Lee Y.J., Lee A., Yoo H.J., Kim M., Noh G.M., Lee J.H. (2018). Supplementation with the probiotic strain *Weissella cibaria* JW15 enhances natural killer cell activity in nondiabetic subjects. J. Funct. Foods.

[34] Nishimura M., Ohkawara T., Tetsuka K., Kawasaki Y., Nakagawa R., Satoh H., Sato Y., Nishihira J. (2015). Effects of yogurt containing *Lactobacillus plantarum* HOKKAIDO on immune function and stress markers. J. Tradit. Complement. Med..

[35] Gill H.S., Rutherfurd K.J., Cross M.L., Gopal P.K. (2001). Enhancement of immunity in the elderly by dietary supplementation with the probiotic *Bifidobacterium lactis* HN019. Am. J. Clin. Nutr..

[36] Miller L.E., Lehtoranta L., Lehtinen M.J. (2017). The Effect of *Bifidobacterium animalis* ssp. lactis HN019 on Cellular Immune Function in Healthy Elderly Subjects: Systematic Review and Meta-Analysis. Nutrients.

[37] Pagnini C., Corleto V.D., Martorelli M., Lanini C., D’Ambra G., Di Giulio E., Delle Fave G. (2018). Mucosal adhesion and anti-inflammatory effects of *Lactobacillus rhamnosus* GG in the human colonic mucosa: A proof-of-concept study. World J. Gastroenterol..

[38] Costabile A., Bergillos-Meca T., Rasinkangas P., Korpela K., de Vos W.M., Gibson G.R. (2017). Effects of Soluble Corn Fiber Alone or in Synbiotic Combination with *Lactobacillus rhamnosus* GG and the Pilus-Deficient Derivative GG-PB12 on Fecal Microbiota, Metabolism, and Markers of Immune Function: A Randomized, Double-Blind, Placebo-Controlled, Crossover Study in Healthy Elderly. Front. Immunol..

[39] Castro-Herrera V.M., Fisk H.L., Wootton M., Lown M., Owen-Jones E., Lau M., Lowe R., Hood K., Gillespie D., Hobbs F.D.R. (2021). Combination of the Probiotics *Lacticaseibacillus rhamnosus* GG and *Bifidobacterium animalis* subsp. *lactis* BB-12 Has Limited Effect on Biomarkers of Immunity and Inflammation in Older People Resident in Care Homes: Results From the Probiotics to Reduce Infections iN CarE home reSidentS Randomized, Controlled Trial. Front. Immunol..

[40] Park H.S. (2023). Lactobacillus rhamnosus LM1019 Strain and Composition for Preventing and Treating Obesity or Diabetes Mellitus Comprising Same. U.S. Patent.

[41] Cho Y.H., Oh S.J. (2010). Casein phosphopeptide-producing activity and proteolytic ability by some lactic acid bacteria. Korean J. Food Sci. Anim. Resour..

[42] You Y., Kim S.H., Kim C.H., Kim I.H., Shin Y., Kim T.R., Sohn M., Park J. (2023). Immune-Stimulating Potential of *Lacticaseibacillus rhamnosus* LM1019 in RAW 264.7 Cells and Immunosuppressed Mice Induced by Cyclophosphamide. Microorganisms.

[43] Cohen S., Kamarck T., Mermelstein R. (1983). A global measure of perceived stress. J. Health Soc. Behav..

[44] Fink L.N., Zeuthen L.H., Christensen H.R., Morandi B., Frøkiaer H., Ferlazzo G. (2007). Distinct gut-derived lactic acid bacteria elicit divergent dendritic cell-mediated NK cell responses. Int. Immunol..

[45] Nagao F., Nakayama M., Muto T., Okumura K. (2000). Effects of a fermented milk drink containing *Lactobacillus casei* strain Shirota on the immune system in healthy human subjects. Biosci. Biotechnol. Biochem..

[46] Foligné B., Zoumpopoulou G., Dewulf J., Ben Younes A., Chareyre F., Sirard J.-C., Pot B., Grangette C. (2007). A key role of dendritic cells in probiotic functionality. PLoS ONE.

[47] Cai S., Kandasamy M., Rahmat J.N., Tham S.M., Bay B.H., Lee Y.K., Mahendran R. (2016). *Lactobacillus rhamnosus* GG activation of dendritic cells and neutrophils depends on the dose and time of exposure. J. Immunol. Res..

[48] Maggini S., Pierre A., Calder P.C. (2018). Immune Function and Micronutrient Requirements Change over the Life Course. Nutrients.

[49] Chandra R.K. (1997). Nutrition and the immune system: An introduction. Am. J. Clin. Nutr..

[50] Li P., Yin Y.L., Li D., Kim S.W., Wu G. (2007). Amino acids and immune function. Br. J. Nutr..

[51] Rolles B., Maywald M., Rink L. (2018). Influence of zinc deficiency and supplementation on NK cell cytotoxicity. J. Funct. Foods.

[52] Amling L., Rink L., Bennstein S.B. (2025). Short-term oral zinc supplementation enhances Natural Killer cell functionality and decreases circulating Innate Lymphoid Cell counts and frequencies in healthy young adults. J. Transl. Med..

[53] Hanson M.G., Özenci V., Carlsten M.C., Glimelius B.L., Frödin J.E., Masucci G., Malmberg K.J., Kiessling R.V. (2007). A short-term dietary supplementation with high doses of vitamin E increases NK cell cytolytic activity in advanced colorectal cancer patients. Cancer Immunol. Immunother..

[54] Lee G.Y., Han S.N. (2018). The Role of Vitamin E in Immunity. Nutrients.

[55] Ha C., Miller L.T., Kerkvliet N.I. (1984). The effect of vitamin B6 deficiency on cytotoxic immune responses of T cells, antibodies, and natural killer cells, and phagocytosis by macrophages. Cell. Immunol..

[56] Schimmer S., Sridhar V., Satan Z., Grebe A., Saad M., Wagner B., Kahlert N., Werner T., Richter D., Dittmer U. (2025). Iron improves the antiviral activity of NK cells. Front. Immunol..

